# Protective Effects of *Cervus elaphus* and *Eucommia ulmoides* Mixture (KGC01CE) on Muscle Loss and Function in Aged Rats

**DOI:** 10.3390/cimb46100664

**Published:** 2024-10-04

**Authors:** Gi-Bang Koo, Han Ol Kwon, Jong Han Kim, Seung Ho Lee, Sung Lye Shim, Kyoung Hwa Jang

**Affiliations:** 1 Laboratory of Efficacy Research, Korea Ginseng Corporation, Gwacheon 13840, Republic of Korea; 20170068@kgc.co.kr (G.-B.K.); jkgm77@kgc.co.kr (H.O.K.); bellone@kgc.co.kr (J.H.K.); fptase@kgc.co.kr (S.H.L.); 2 Laboratory of Resource and Analysis, Korea Ginseng Corporation, Gwacheon 13840, Republic of Korea; shimsl@kgc.co.kr

**Keywords:** sarcopenia, muscle atrophy, *Cervus elaphus*, *Eucommia ulmoides*, KGC01CE

## Abstract

Sarcopenia is a condition characterized by a progressive loss of muscle mass and function which are influenced by certain factors such as aging, nutritional deficiencies, and chronic diseases. Despite numerous efforts to prevent or treat sarcopenia, effective therapeutic options for this disease remain limited. This study aims to evaluate the effects of KGC01CE treatment, a mixture of *Cervus elaphus* (Ce) and *Eucommia ulmoides* (Eu), which are well-known traditional herbal medicines in Asia, on age-related muscle loss and functional decline in aged rats. KGC01CE has been found to be more effective than the individual extracts in inhibiting dexamethasone (DEX)-induced muscle atrophy and improving muscle mass and grip strength in C2C12 cells and aged rats. Moreover, animal studies were conducted to determine the minimum effective dose, and a 12-week oral administration of KGC01CE treatment at doses of 50, 100, and 200 mg/kg to 15-month-old aged rats resulted in a dose-dependent increase in lean mass, muscle mass, grip strength, and muscle cross-sectional area (CSA), which had decreased due to aging. Furthermore, it was shown that KGC01CE activated the phosphatidylinositol 3-kinase (PI3K)/Akt pathway and inhibited the expression of muscle-degrading proteins MuRF, Atrogin-1, and myostatin. These results suggest that KGC01CE treatment may effectively prevent muscle loss and functional decline, providing a novel therapeutic strategy for sarcopenia.

## 1. Introduction

Sarcopenia is characterized by a decrease in muscle mass, strength, and function and is influenced by certain factors, such as aging, decreased nutritional intake, inflammation, chronic disease, and hypokinesia [1,2]. Patients with sarcopenia tend to be vulnerable to impairments in daily living activities and quality of life, diverse types of diseases, and higher mortality. Moreover, decreased muscle mass may also cause bone health impairments and metabolic dysfunction, and its association with metabolic diseases, such as obesity and diabetes mellitus, infections, and cancer has also been investigated in numerous studies [3,4,5,6,7,8]. It was previously considered a natural, aging-related phenomenon. Recently, however, a widespread consensus on its seriousness has been reached; it has also been assigned with the disease code ICD-10-CM (M62.84) in accordance with the World Health Organization (WHO) recommendations [9].

To identify the effective therapeutic treatments for sarcopenia, molecular biological studies using diverse sarcopenia models, such as an aging model, drug-induced model, nerve dissection model, immobilization model, and disease model (e.g., obesity, DM, and cachexia), have been conducted [10,11,12]. Such studies have suggested that sarcopenia is caused by increased apoptosis, an imbalance between protein synthesis and degradation, oxidative stress, inflammatory responses, hormonal changes, and decreased muscle regeneration [13,14]. Decreased muscle mass is a key feature of sarcopenia, with the inhibition of protein synthesis and increased protein degradation being the primary mechanisms. Considering that multiple protein degradation pathways exist, including the autophagy–lysosome, calcium-dependent calpain, and cysteine aspartate protease systems, the ubiquitin–proteasome pathway emerges a cardinal avenue for intracellular protein degradation, suggesting a significant influence on the muscle atrophy trajectory [15]. Of note, the increased levels of representative E3 ubiquitin ligases, such as muscle atrophy F-box (Atrogin-1/MAFbx) and muscle RING finger-1 (MuRF1), are associated with reduced muscle mass. Myostatin is a key protein associated with muscle inhibition, and its levels have been shown to be elevated in various sarcopenia disease models, including aged rats [16,17,18]. Mice that lack myostatin exhibit muscle hypertrophy, which is characterized by increased muscle mass. Myostatin is known to inhibit the Akt signaling pathway, which is crucial for muscle protein synthesis, thereby suppressing skeletal muscle hypertrophy [19]. A representative muscle synthesis signaling pathway is the PI3K/Akt signaling pathway. In the skeletal muscle, this pathway activates the mammalian target of rapamycin (mTOR)/p70S6K signaling cascade, promoting protein synthesis and increasing muscle mass through inhibiting the expression of atrogin-1. Moreover, the major molecular causes of sarcopenia include inflammatory responses, oxidative stress, and hormonal changes. Although nutritional supplements, hormonal therapies (e.g., IGF-1), exercise therapy, drug treatments (e.g., myostatin inhibitors), and anti-inflammatory agents have been used for the management of sarcopenia, no specific therapeutic agents for its treatment have been approved to date. Current recommendations for preventing sarcopenia have emphasized the intake of essential amino acids, resistance exercise, and aerobic exercise. However, these strategies are limited, which necessitates new treatment approaches that are safe, effective, and applicable to everyone.

*Cervus elaphus* (Ce) has been traditionally used to prevent and treat diverse diseases in several countries including East Asia. It has been shown to exhibit immunological, anti-fatigue, and anti-arthritic effects [20,21]. Moreover, its major constituents include sialic acid, free amino acids, prostaglandins, and gangliosides [22]. *Eucommia ulmoides* (Eu) is mainly found in East Asia; its bark and leaf have been commonly used as an herbal treatment [23,24]. Its pharmacological characteristics include anti-bacterial [25], antioxidative [26], anti-hyperlipidemic [27], anti-inflammatory [28,29], anti-obesity [30], anti-diabetic [31], anti-hypertensive [32], anti-renal injury [33], and neuroprotective effects [34]. Moreover, its major constituents comprise diverse chemicals including lignans, iridoids, phenols, steroids, and flavonoids [35].

This study aims to evaluate the effects of KGC01CE treatment on sarcopenia. To determine the optimal ratio of these extracts, muscle atrophy, muscle mass, and grip strength were assessed using a DEX-induced muscle atrophy cell model and aged rats. Furthermore, to evaluate the minimum effective concentration of the optimal combination of KGC01CE, various concentrations were orally administered to aged rats and changes in lean mass, muscle mass, grip strength, muscle CSA, and underlying mechanisms were analyzed. These results could confirm the use of dietary supplements for alleviating and preventing sarcopenia.

## 2. Materials and Methods

### 2.1. Preparation of KGC01CE

KGC01CE is a powdered extract complex produced via combining the water extract concentrate of Ce and the 30% aqueous ethanol extract concentrate of Eu, followed by spray drying. The Ce extract concentrate was prepared using New Zealand-sourced *Cervus elaphus* via two successive extractions with distilled water at temperature of 87 °C for 6 h each, followed by concentration until the solid content reached 20%. Eu extract concentrate was prepared using the bark of *Eucommia ulmoides* via two successive extractions with 30% ethanol at a temperature of 80 °C for 10 h each and then concentrated until the solid content reached 20%. This extract was used as a standard sample for all efficacy evaluations. Ce refers to a single extract of *Cervus elaphus*, while Eu refers to a single extract of *Eucommia ulmoides*. The capitalized CE denotes a mixture of *Cervus elaphus* and *Eucommia ulmoides*. KGC01CE specifically refers to a 1:3 mixture of *Cervus elaphus* and *Eucommia ulmoides*.

### 2.2. Standardization of KGC01CE

Sialic acid and pinoresinol diglucoside, which are representative compounds of the raw materials, were selected as the marker compounds, and the complex was standardized based on their content [22,35,36]. Sialic acid, a key marker of the *C. elaphus* extract, was analyzed using the high-performance liquid chromatography with Fluorescence detector (HPLC-FLD) system. Five hundred mg of KGC01CE powder was transferred into a 50 mL volumetric flask and 45 mL of 20 mM HCl was added; the mixture was shaken well and extracted using an ultrasonic system (60 Hz; Wiseclean, Seoul, Republic of Korea) for 30 min. An acid hydrolysis was performed at 85 °C for 1 h and cooled, and the volume was adjusted to 50 mL with additional solution. The extract was centrifuge and syringe-filtered to remove the residues. Fluorescence labeling was performed in accordance with the protocol provided with the sialic acid fluorescence labeling kit (Takara Cat# 4400). HPLC analysis was performed using the HPLC-FLD system (Waters Co., Milford, MA, USA) equipped with a YMC-Pack NH2 column (5.0 µm, 4.6 × 250 mm). A gradient mobile phase with distilled water (solvent A) and acetonitrile (solvent B), both containing 0.1% formic acid, was used. The gradient program was as follows: 0–3 min (5% A), 4–16 min (13% A), 18–20 min (50% A), and 22–25 min (5% A).

Pinoresinol diglucoside, a key marker of the *E. ulmoides* extract, was prepared and analyzed using the HPLC system. Two hundred mg of KGC01CE was transferred into a 50 mL volumetric flask and extracted twice with 40 mL of 30% aqueous methanol for 30 min under an ultrasonic system. The volume was adjusted to 50 mL with additional solution, centrifuged, and syringe-filtered to remove the residues. A HPLC analysis was performed using an HPLC system equipped with a CAPCELL PAK ADMK column (3.0 µm, 4.6 × 150 mm). A gradient mobile phase with 0.1% formic acid in distilled water (solvent A) and 0.1% formic acid in acetonitrile (solvent B) was used. The gradient program was as follows: 0 min (95% A), 5 min (90% A), 10 min (85% A), 15 min (80% A), 20 min (75% A), 25 min (70% A), 30 min (5% A), and 31–35 min (95% A).

### 2.3. Reagent

Primary antibodies, including phospho-PI3K, phospho-Akt, and glyceraldehyde-3-phosphate dehydrogenase (GAPDH), were obtained from Cell Signaling Technology (Danvers, MA, USA). Myostatin, atrogin-1, and MuRF were obtained from Santa Cruz Biotechnology (Santa Cruz, CA, USA). Dexamethasone (DEX) and oxymetholone were purchased from Sigma-Aldrich (St. Louis, MO, USA). Moreover, isoflurane (KNOTUS Co. Ltd., Incheon, Republic of Korea) was used to induce anesthesia in laboratory animals.

### 2.4. Cell Culture and Treatment

Murine C2C12 myoblasts were acquired from ATCC. The cells were cultured in growth media consisting of 90% Dulbecco’s Modified Eagle Medium (DMEM), 10% fetal bovine serum (FBS), and 100 units/mL penicillin-streptomycin (PS) at a temperature of 37 °C with 5% CO_2_. To induce differentiation, C2C12 myoblasts were grown to over 90% confluency. The growth medium was then replaced every 48 h with differentiation medium containing 90% DMEM, 10% horse serum, and 100 units/mL PS, and the cells were allowed to differentiate for 6 days. Following differentiation, the cells were pre-treated for 1 h with Ce, Eu, and CE (with ratios of 3:1, 1:1, or 1:3), and then treated with DEX. After 24 h of DEX treatment, the myotube diameters were analyzed using the ImageJ software 1.53e, with observations made through an inverted microscope (Olympus IX81; Olympus, Tokyo, Japan).

### 2.5. Animal Experiment

For the ratio optimization experiment, 35 male SD rats aged 15 months and 5 male SD rats aged 3 months were obtained from KOATECH (Pyeongtaek-si, Gyeonggi-do, Republic of Korea) and acclimated in the animal housing facility for 2 weeks. After acclimation, the rats were randomly assigned into groups according to body weight: young SD rat (NC), aged SD rat (C), aged SD rat + oxymetholone (PC), aged SD rat + Ce (Ce), aged SD rat + Eu (Eu), aged SD rat + Ce:Eu (3:1, CE(3:1)), aged SD rat + Ce:Eu (1:1, (CE1:1)), and aged SD rat + Ce:Eu (1:3, (CE1:3)), with 5 rats per group. During the 12-week treatment period, the test substances were orally administered at a dose of 100 mg/kg once daily. The PC group was orally administered with oxymetholone a dose of 10 mg/kg simultaneously [37]. After the 12-week treatment period, the grip strength, and gastrocnemius (GAS) muscle weight were measured to determine the optimal ratio.

To establish the minimum effective dose, 24 15-month-oldmale SD rats and 6 3-month-old male SD rats were obtained and acclimated for 2 weeks. After acclimation, the rats were randomly assigned into groups according to body weight: NC, C, *C.elaphus:E.ulmoides* (1:3, KGC01CE) at doses of 50 mg/kg (KGC01CE-50), 100 mg/kg (KGC01CE-100), and 200 mg/kg (KGC01CE-200) groups, with 6 rats per group. During the 12-week treatment period, KGC01CE was orally administered at doses of 50, 100, and 200 mg/kg once daily. After the 12-week treatment period, lean mass, grip strength, and muscle mass were measured. The rats were anesthetized with isoflurane for the muscles (e.g., GAS, soleus muscle [SOL], tibialis anterior muscle [TA], extensor digitorum longus [EDL]), and serum collection was performed. The serum was analyzed for biochemical markers (e.g., AST, ALT, CREA, BUN) while the muscle tissues were weighed, sectioned, and analyzed for protein content and muscle CSA via protein extraction and H&E staining.

All the animal experiments were approved by the Institutional Animal Care and Use Committee (IACUC) of Korea Ginseng Corporation (IACUC approval # KGC-2020-023 and -007). The condition of the animal facility was maintained at a temperature of 22 ± 2 °C, relative humidity of 50 ± 10%, ventilation frequency of 10–15 times/h, light exposure for 12 h (8 AM–8 PM), and light intensity of 250–300 Lux. The animals were provided with ad libitum access to food and water, and their body weight and diet were monitored weekly.

### 2.6. Dual Energy X-ray Absorptionmetry Analysis

The muscle mass of the SD rats was measured using a dual-energy X-ray absorptiometry (DXA) scanner (iNSiGHT VET DXA; Osteosys, Seoul, Republic of Korea). Before autopsy, they were anesthetized using isoflurane (Piramal Critical Care Inc., Bethlehem, PA, USA) and were in the prone position on the imaging plate with their legs and tails extended maximally. A cone beam X-ray, ranging from low energy (60 kV/0.8 mA) to high energy (80 kV/0.8 mA), was used to capture high-resolution images using a flat panel detector. The images were then analyzed using the iNSiGHT software version 1.0.6 (Osteosys).

### 2.7. Grip Strength Test

After a 12-week treatment, the muscle strength was measured using a grip strength meter (JD-A-22; Jeungdo Bio & Plant Co., Ltd., Seoul, Republic of Korea). For the measurement, the forelimbs of the SD rats were placed on the grid, while the body trunk was held at body height and the tail was pulled with consistent force. The measurements were performed three times per animal, and the results were averaged.

### 2.8. CSA Analysis

To conduct the CSA analysis of the muscle fibers, the medial part of the GAS was dissected and then fixed in a 10% formalin solution for 24 h and embedded in paraffin. The cross-sectional slides were prepared at a thickness of 3 µm and then stained using H&E dye in an automated equipment (Tissue-TekPrisma^®^ Plus Automated Slide Stainer; SakuraTM, Tokyo, Japan). The images were captured at a magnification rate of ×100 using an inverted microscope (Olympus BX51 microscope) (Olympus Co. Ltd., Tokyo, Japan) and then analyzed using the Image J software version 1.49 (National Institutes of Health, Bethesda, MD, USA). The mean value of the CSA was quantified relative to the sham group set at 100%.

### 2.9. Protein Extraction and Western Blotting Analysis

To analyze the protein, the medial part of the GAS muscle was collected at a weight of 50 mg and then homogenized in a radioimmune precipitation assay (RIPA) buffer (Biosesang Inc., Yongin, Republic of Korea). After the homogenized solution was centrifuged, the supernatant containing the protein was harvested and then quantified equally using the Bradford reagent (Sigma-Aldrich, St. Louis, MO, USA). The same amount of protein was separated using 4–12% sodium dodecyl sulfate-polyacrylamide gel electrophoresis (SDS-PAGE) (Bolt Bis-Tris Plus gels; Thermo Fisher Scientific, Waltham, MA, USA) and a polyvinylidene fluoride (PVDF) membrane. After blocking, the membrane was incubated with primary antibodies (1:1000) for over 12 h. Moreover, the sample was rinsed using phosphate-buffered saline with Tween20 (PBST) (20X TBS Tween-20 buffer; Thermo Fisher Scientific, Waltham, MA, USA) and then reacted with the secondary antibodies at room temperature for 1 h. After the sample was rinsed using PBST three times, the images were obtained using the Davinch equipment in accordance with the instructions of the manufacturer of enhanced chemiluminescence (ECL).

### 2.10. Serum Biochemistry

The serum samples were collected from the SD rats and then assayed using the Hitachi Automatic Biochemical Analyzer 7180 (Hitachi, Yokohama, Japan). Thus, the current study measured ALT/AST, which serve as the hepatic parameters, and creatine and BUN, which serve as the renal parameters.

### 2.11. Statistical Analysis

All the experimental results were analyzed using the GraphPad Prism software (version 10.3.1; GraphPad Software, San Diego, CA, USA). Data were expressed as mean ± standard deviation (SD). The significant differences between the experimental groups were assessed using the analysis of variance, Student’s *t*-test, and Tukey’s multiple comparison test. Statistical significance was set at a *p*-value of <0.05.

## 3. Results

### 3.1. Preparation and Standardization of KGC01CE

**Figure 1:**
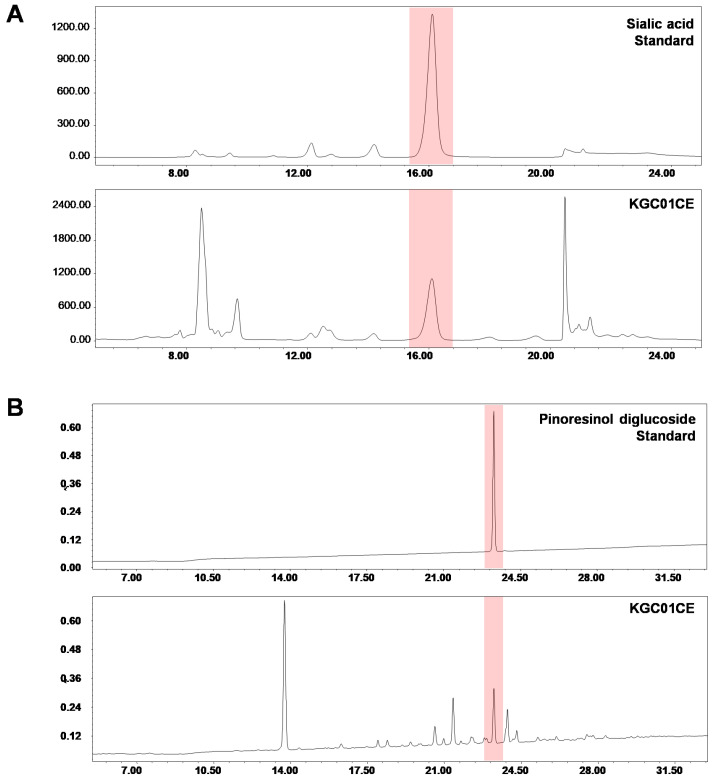
Representative HPLC chromatograms of sialic acid and pinoresinol diglucoside in KGC01CE (Ce:Eu= 1:3). (**A**) HPLC chromatograms indicating the sialic acid standard and sialic acid content in KGC01CE. (**B**) HPLC chromatograms indicating the pinoresinol diglucoside standard and pinoresinol diglucoside content in KGC01CE.

KGC01CE is a powdered extract blend consisting of Ce and Eu with a 1:3 ratio. Ce was extracted twice with distilled water at a temperature of 87 °C for 6 h each, filtered, and concentrated under reduced pressure to obtain a 20% solid concentrate. Eu was extracted twice with 30% ethanol at a temperature of 80 °C for 10 h each, filtered, and concentrated to a 20% solid concentrate. Moreover, the extracts were combined with a 1:3 ratio, mixed with 15% dextrin, and spray-dried to produce KGC01CE powder. The standardization of KGC01CE was achieved based on sialic acid and pinoresinol diglucoside content. The HPLC analysis quantified the sialic acid at 0.40 mg/g and pinoresinol diglucoside at 14.8 mg/g (Figure 1).

### 3.2. Optimization of the Mixing Ratio of KGC01CE in DEX-Induced C2C12 Myotube Atrophy and Aged Rats

**Figure 2:**
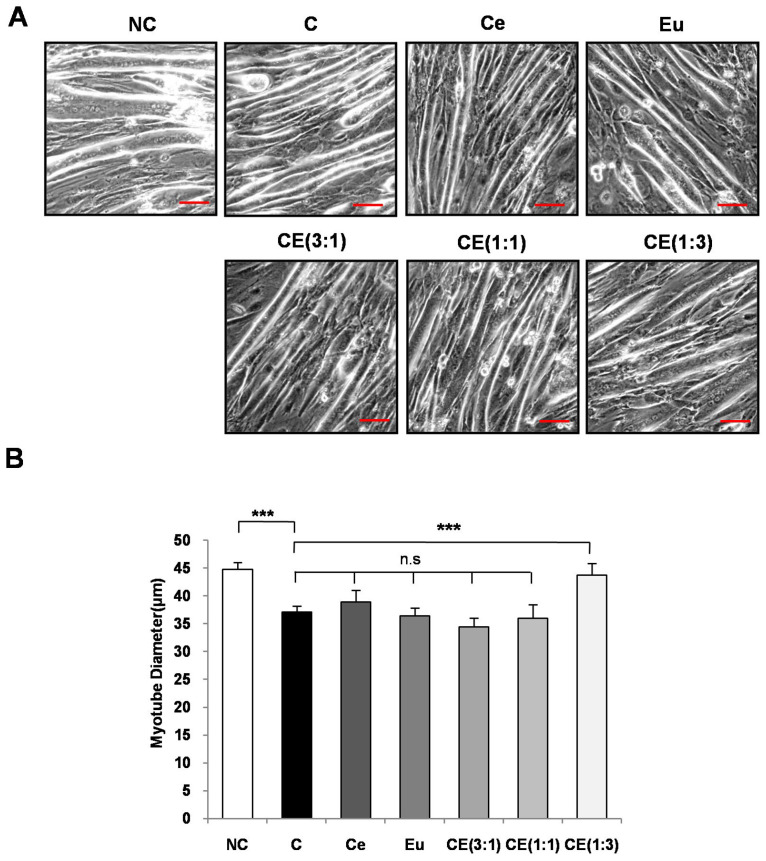
Effect of KGC01CE on DEX-induced muscle atrophy in the C2C12 models. (**A**) Representative image of muscle atrophy. (**B**) Myotube diameter. After pre-treatment with 50 μg/mL of individual extracts or a ratio-based mixture in differentiated myotubes, 100 μm DEX treatment was administered for 24 h. Subsequently, the images were captured using a microscope, and the myotube diameter was measured using the ImageJ software. NC: negative control (untreated), C:DEX, Ce: Ce + DEX, Eu: Eu+ DEX, CE(3:1): Ce:Eu (3:1)+ DEX, CE(1:1): Ce:Eu (1:1) + DEX, CE(1:3): Ce:Eu (1:3) + DEX. Data are expressed as mean ± SD. *** *p*< 0.001, n.s: not significant, scale bar = 50 μm.

To determine the optimal ratio of Ce and Eu mixture for mitigating muscle atrophy, we induced muscle atrophy in myotubes using DEX, which is known to induce muscle atrophy through activating muscle-degrading proteins such as MuRF and atrogin-1 [38]. Compared with the NC group, the C group treated with DEX exhibited an 18% reduction in myotube diameter. At a non-toxic concentration of 50 μg/mL, Eu extract alone reduced muscle atrophy by 23.4%, whereas Ce extract alone exhibited no significant effect. Of the various ratios tested, the CE(1:3) treatment indicated the highest degree of inhibition (Figure 2).

To validate these cellular findings using an in vivo model, experiments were performed using aged SD rats. They were administered with either Ce, Eu, or their combinations CE(3:1, 1:1, 1:3) at a constant dose of 100 mg/kg orally for 12 weeks (Figure S1A). Post-treatment, muscle mass in the GAS muscle and grip strength were assessed. Consistent with the in vitro data, the C group showed a significant decrease in both muscle mass and grip strength compared with the NC group. In contrast, CE(1:3) significantly enhanced muscle mass and grip strength more effectively than the other ratios tested (Figure S1B,C). These findings suggest that the CE(1:3) treatment provides the optimal efficacy in mitigating muscle atrophy.

### 3.3. KGC01CE Increased Muscle Weight in Aged Rats

**Figure 3:**
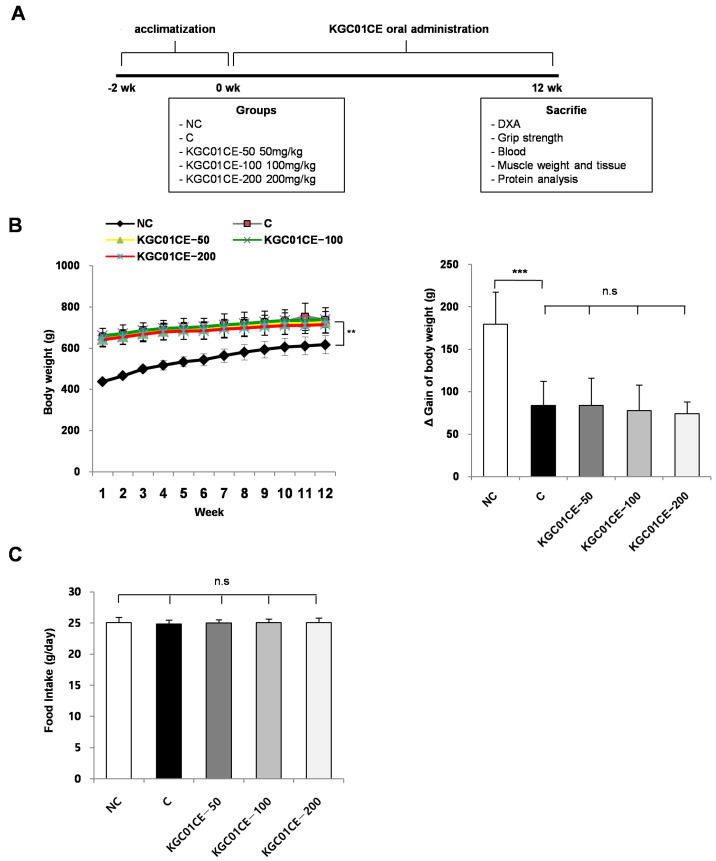
Effect of KGC01CE on body weight and food intake in aged rats. (**A**) Experimental design, (**B**) body weight, and (**C**) food intake (g/day). Fifteen-month-old male rats were orally administered with KGC01CE at concentrations of 50, 100, and 200 mg/kg for 12 weeks. NC: young SD rats, C: aged SD rats, KGC01CE − 50:aged SD rats + CE(1:3) 50 mg/kg b.w, KGC01CE − 100:aged SD rats +CE(1:3) 100 mg/kg b.w, KGC01CE − 200: aged SD rats +CE(1:3) 200 mg/kg b.w. Data are expressed as mean ± SD (*n* = 6). ** *p* < 0.01, *** *p* < 0.001, n.s: not significant.

To determine the minimum effective concentration of the KGC01CE for inhibiting muscle loss, the aged rats were orally administered with varying concentrations of the complex for 12 weeks (Table 1). The KGC01CE was administered at concentrations of 50, 100, and 200 mg/kg (Figure 3A). The administration of KGC01CE did not significantly affect body weight or food intake (Figure 3B,C). Although a trend toward decreased body weight gain with increasing concentrations was observed, the changes were not statistically significant (Figure 3B).

**Figure 4:**
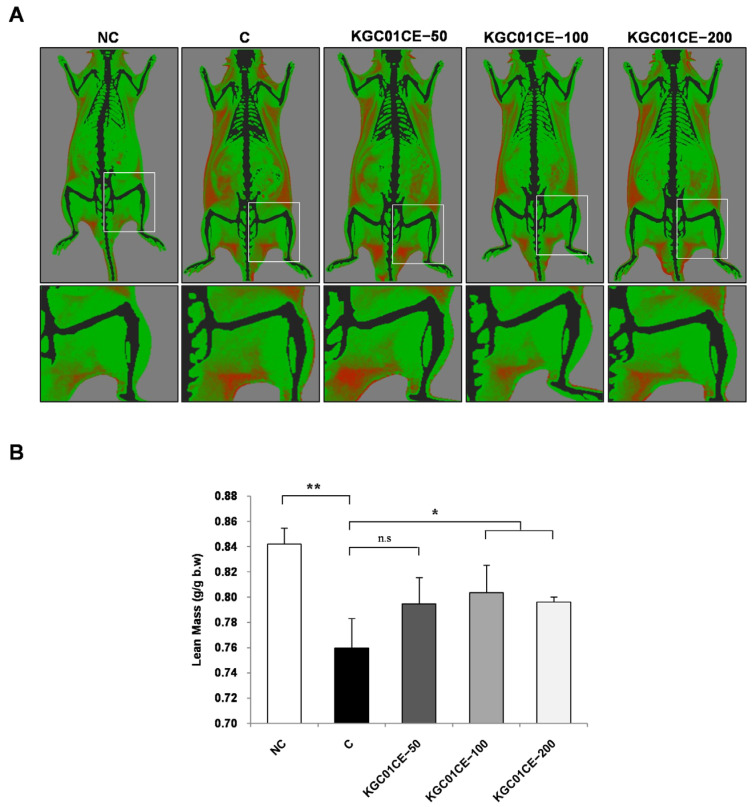
Effect of KGC01CE on lean mass in aged rats. (**A**) Representative image from DXA, (**B**) lean mass measured using DXA. After 12 weeks of administration, lean mass was assessed using DXA. Total lean mass was expressed as a ratio to body weight. NC: young SD rats, C: aged SD rats, KGC01CE − 50: aged SD rats + CE(1:3) 50 mg/kg b.w, KGC01CE − 100: aged SD rats + CE(1:3) 100 mg/kg b.w, KGC01CE − 200: aged SD rats + CE(1:3) 200 mg/kg b.w. Data are expressed as mean ± SD (*n* = 4). * *p*< 0.05, ** *p*< 0.01, n.s: not significant.

Furthermore, no significant differences in the safety indicators for liver and kidney function were observed when compared with the C group, indicating the safety of KGC01CE treatment (Table 2). After 12 weeks of treatment, the total lean mass was measured using DXA. Compared with the NC group, the C group showed a decrease in total lean mass in the hind limbs (Figure 4A). In contrast, treatment with the KGC01CE at various concentrations resulted in an increased lean mass relative to body weight (Figure 4B).

**Figure 5:**
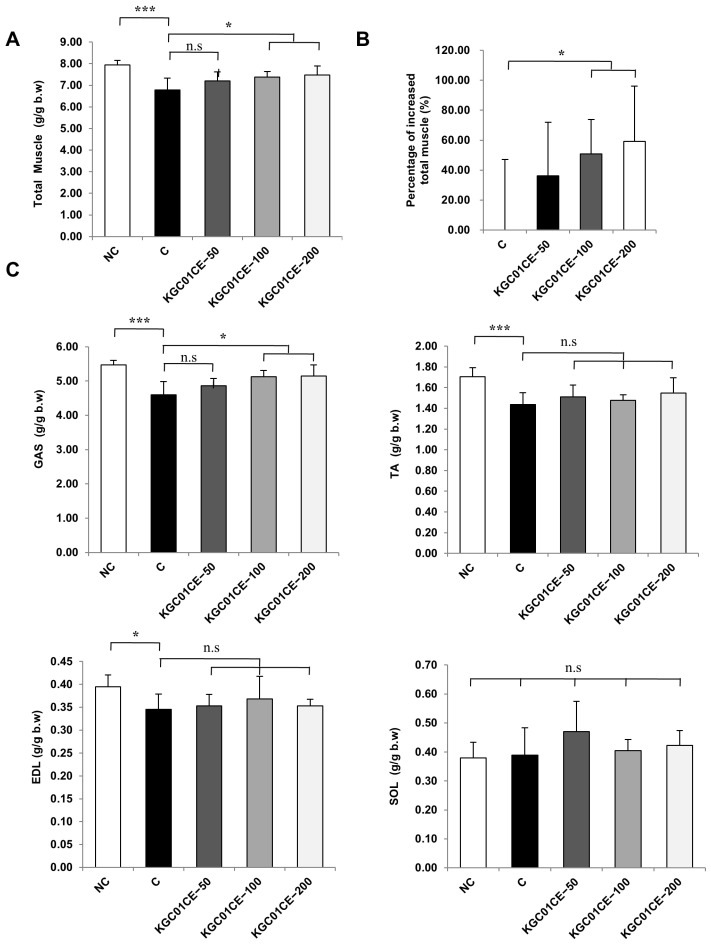
Effect of KGC01CE on muscle mass in aged rats. (**A**) Total muscle mass (sum of the GAS, TA, EDL, and SOL muscles), (**B**) percentage of increased muscle, and (**C**) weight of the GAS, TA, EDL, and SOL. Muscle weight expressed as a ratio of muscle weight to body weight (g/g). NC: young SD rats, C: aged SD rats, KGC01CE − 50: aged SD rats + CE(1:3) 50 mg/kg b.w, KGC01CE − 100: aged SD rats + CE(1:3) 100 mg/kg b.w, KGC01CE − 200: aged SD rats + CE(1:3) 200 mg/kg b.w. Data are expressed as mean ± SD (*n* = 6). * *p* < 0.05, *** *p* < 0.001, n.s: not significant.

To confirm the increase in individual muscle mass, four muscles from the hind limbs (GAS, TA, EDL, and SOL muscles) were dissected and weighed. The total muscle mass (sum of GAS, TA, EDL, and SOL) was significantly lower in the C group than in the NC group, whereas the KGC01CE treatment group showed concentration-dependent increases of 36%, 50%, and 59% in the total muscle mass compared to the C group, relative to the NC group (*p* <0.05) (Figure 5A,B). As shown in Figure 5C, the measurements of individual muscle mass following KGC01CE treatment revealed a significant, concentration-dependent increase in the mass of the GAS muscle. Moreover, although not statistically significant, increases were observed in TA, EDL, and SOL muscles following KGC01CE treatment. These results suggested that KGC01CE effectively mitigates muscle loss associated with aging.

### 3.4. KGC01CE Significantly Increased the Grip Strength and Muscle CSA in Aged Rats

**Figure 6:**
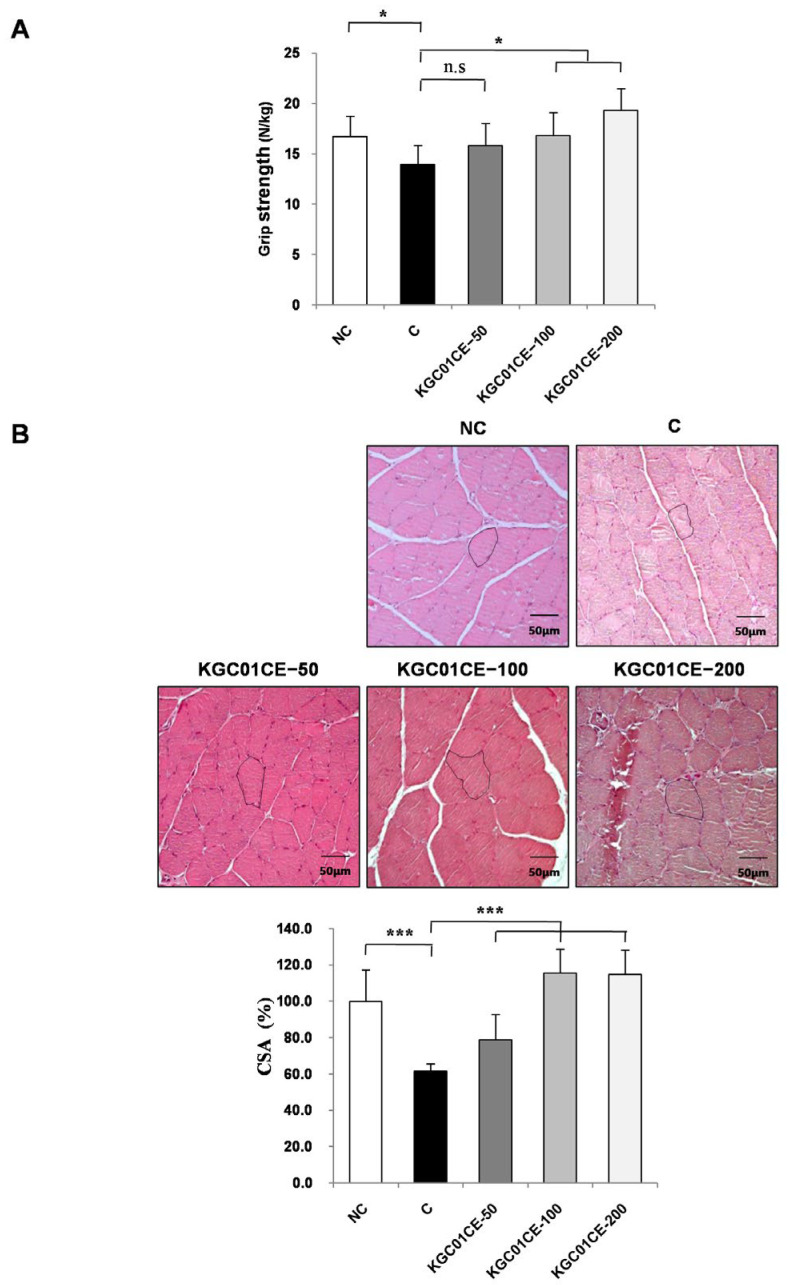
Effect of KGC01CE on grip strength and CSA in aged rats. (**A**) Grip strength (N/kg), (**B**) representative H&E image, (**C**) mean of CSA. Grip strength was assessed after 12 weeks of administration and expressed as a grip strength to body weight ratio. CSA was expressed as a percentage relative to the NC value. NC: young SD rats, C: aged SD rats, KGC01CE − 50: aged SD rats + CE(1:3) 50 mg/kg b.w, KGC01CE − 100: aged SD rats + CE(1:3) 100 mg/kg b.w, KGC01CE − 200: aged SD rats + CE(1:3) 200 mg/kg b.w. Data are expressed as mean ± SD (*n* = 6). * *p* < 0.05, *** *p* < 0.001, n.s: not significant.

A decrease in both muscle mass and strength is a hallmark symptom of sarcopenia. Grip strength is a widely used indicator for evaluating sarcopenia that serves as a proxy for overall physical strength and muscle function [39]. To assess the effect of KGC01CE on age-related decline in grip strength, forelimb grip strength was measured over a 12-week period. The C group showed a significant 16.7% reduction in grip strength (13.9 N/kg) compared with the NC group (16.7 N/kg) (*p* < 0.05). However, the aged rats administered with KGC01CE showed a concentration-dependent increase in grip strength over the 12-week treatment period (Figure 6A). Significantly, at concentrations of 100 and 200 mg/kg, the grip strength significantly improved compared to the C group (*p* < 0.05).

Muscle CSA is known to decrease with aging, and the correlation between CSA and muscle strength has been well-established. To validate the increase in muscle strength due to KGC01CE administration and to evaluate muscle quality, the CSA of the GAS muscle fibers was assessed through H&E staining. The average CSA in the C group (aged rats) was a significant 38% lower than in the NC control group (*p* < 0.05). In contrast, the administration of KGC01CE resulted in a concentration-dependent increase in CSA compared with the C group (50, 100, and 200 mg/kg, *p* < 0.05). Specifically, at 100 and 200 mg/kg, CSA was restored to levels comparable with the NC group (Figure 6B). These results suggested that the KGC01CE treatment effectively increased the muscle CSA, thereby significantly improving the grip strength.

### 3.5. KGC01CE Regulates the Up-Regulation of the PI3K-Akt Pathway and the Down-Regulation of MuRF-1, atrogin-1, and Myostatin in Aged Rats

**Figure 7:**
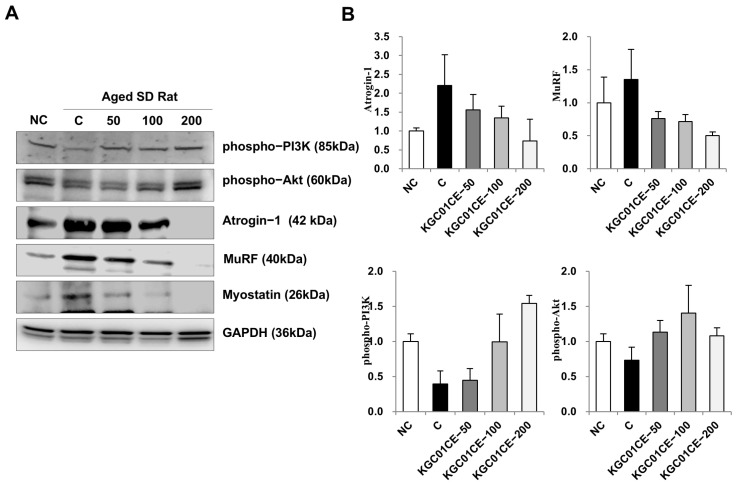
Effect of KGC01CE on muscle protein synthesis and degradation pathway in aged rats. (**A**) Representative Western blot image of phospho-PI3K, phospho-Akt, MuRF-1, atrogin-1, and myostatin. (**B**) The protein expression levels were normalized to the GAPDH. The proteins extracted from the gastrocnemius muscle were analyzed using Western blotting. The PI3K/Akt signaling pathway for muscle protein synthesis was assessed, and the MuRF-1, atrogin, and myostatin related to muscle breakdown were evaluated. Quantification was performed relative to GAPDH using the ImageJ software and the results were expressed as fold change normalized to the NC.NC: young SD rats, C: aged SD rats, KGC01CE − 50: aged SD rats + CE(1:3) 50 mg/kg b.w, KGC01CE − 100: aged SD rats + CE(1:3) 100 mg/kg b.w, KGC01CE − 200: aged SD rats + CE(1:3) 200 mg/kg b.w.

To analyze the molecular mechanisms underlying the increase in muscle mass, grip strength, and muscle CSA induced by the KGC01CE treatment, the proteins associated with muscle protein synthesis (PI3K/Akt) and degradation (MuRF-1, atrogin-1, myostatin) were assessed. Muscle mass is increased through the PI3K/Akt signaling pathway, while degradation is mediated by MuRF-1 and atrogin-1 [13,15]. Myostatin, a negative regulator of muscle growth, inhibits muscle protein synthesis via suppressing the Akt signaling [17]. The analysis of proteins extracted from the GAS muscle revealed that the administration of the KGC01CE resulted in a concentration-dependent increase in PI3K/Akt signaling compared with the C group (Figure 7A,B). Conversely, the expression of muscle degradation proteins atrogin-1 and MuRF-1, which are E3 ubiquitin ligases, was reduced in the KGC01CE treatment group (Figure 7A,B). Moreover, myostatin, which is known for its role in promoting muscle degradation and as a target for various therapeutic interventions, has shown a concentration-dependent decrease in expression following KGC01CE treatment (Figure 7A,B). These findings collectively indicate that KGC01CE effectively enhances muscle protein synthesis through up-regulating the PI3K/Akt pathway and concurrently inhibits muscle degradation via the down-regulation of MuRF-1, atrogin-1, and myostatin. Consequently, KGC01CE can contribute to mitigating muscle mass loss and dysfunction through increasing muscle protein synthesis and inhibiting protein degradation.

## 4. Discussion

The primary treatments for sarcopenia include resistance exercise and nutritional support; however, no specific drugs for the treatment of this condition have been approved. Although various options, such as hormonal therapies, growth hormones, muscle protein breakdown inhibitors, vitamins, and nutritional supplements, have been developed for the potential treatment of sarcopenia, finding a safe and effective therapy for this disease is challenging due to the side effects of the treatments and the multifactorial nature of the disease.

This study aimed to identify the potential agents for preventing or alleviating sarcopenia from traditionally used, safe sources. Ce extract and Eu extract are widely used traditional remedies in East Asia. They are traditionally known for their benefits in relieving muscle fatigue, enhancing physical strength, promoting muscle growth, and providing general tonic effects. The optimal combination and concentration of KGC01CE treatment for preventing and alleviating sarcopenia based on traditional evidence was determined.

In this study, we confirmed that a 1:3 ratio of Ce to Eu tends to produce and optimal synergistic effect. To evaluate the synergistic effects of Ce and Eu extracts, we assessed the muscle atrophy in C2C12 mouse muscle cell using DEX, which is known to induce muscle atrophy through inhibiting the PI3K/Akt signaling pathway and activating muscle protein breakdown enzymes such as atrogin-1 and MuRF-1. DEX significantly reduced the diameter of differentiated muscle cells. However, the CE(1:3) significantly counteracted this reduction in cell diameter compared with the C group, single-agent groups, and mixed groups (3:1, 1:1).Furthermore, to validate these cell-based results in an animal model, aged rats were orally administered with either the individual extracts or the CE treatment at a dosage of 100 mg/kg for 12 weeks. The muscle mass (GAS) and grip strength, the key indicators of sarcopenia, were measured. In a previous study, aged rats showed significant reductions in muscle mass (e.g., GAS, TA, EDL, and SOL muscles)and strength [40]. In contrast, the CE(1:3) significantly increased muscle mass (GAS) and grip strength compared with the C, CE(3:1), and CE(1:1) groups, suggesting that this mixture is optimal for preventing or alleviating sarcopenia.

To determine the MEC of the KGC01CE treatment in animal experiments, aged rats were orally administered with doses of 50, 100, and 200 mg/kg/day for 12 weeks while the lean mass, muscle mass, grip strength, and muscle fiber CSA were evaluated. DXA data indicated that the C group (aged rats) had a significant 10% reduction in lean mass compared with the NC group (young rats). In contrast, the treatment groups exhibited increasing trends of 42.7%, 53.3%, and 44.1% in lean mass, respectively, when the difference between the NC group and the C group was calculated as 100%. To further validate the DXA results and assess muscle mass through muscle type, individual muscles were dissected and weighed. Consistent with the DXA results, the C group (aged rats) showed a significant 14.8% reduction in the total weight of four muscles (GAS, TA, EDL, and SOL muscles) compared with the young rats. However, KGC01CE significantly increased the muscle mass at all doses in total muscle and GAS, with notable increases in the GAS muscle at 100 mg/kg and 200 mg/kg doses. The GAS muscle plays a central role in lower limb functionality, which is crucial for physical abilities and daily activities. To assess the functional impact of increased muscle mass, the grip strength and muscle fiber CSA were measured. Grip strength is a valid indicator of muscle strength, and an increase in muscle fiber CSA is associated with enhanced strength. While the grip strength and muscle fiber CSA were significantly reduced in the C group (aged rats) compared with the NC group (young rats), both parameters were increased in the KGC01CE treatment group in a dose-dependent manner. These results indicate that the MEC of KGC01CE is 100 mg/kg or higher and suggest that KGC01CE treatment is effective in mitigating age-related muscle loss, strength reduction, and the decrease in muscle fiber CSA.

Among the various molecular biological causes of sarcopenia, the most representative is the imbalance between protein synthesis and degradation. Various factors such as oxidative stress, inflammation, hormonal imbalances, and nutrition ultimately lead to an imbalance in protein synthesis and degradation, which can result in loss of muscle mass and function. To determine the mechanism of KGC01CE in relation to its muscle-protective effects, the key proteins related to muscle protein synthesis (PI3K/Akt) and degradation (MuRF-1, atrogin-1, myostatin) in the GAS muscle were analyzed, which indicated a significant muscle mass increase. Based on the molecular biology of muscle wasting, the balance between muscle synthesis and degradation pathways is crucial for muscle maintenance. Some studies have indicated that in aging rats, the PI3K/Akt signaling pathway, which is essential for muscle synthesis, decreased, while the expression of the degrading proteins (MuRF, atrogin-1, and myostatin) increased [41,42]. This imbalance in synthesis and degradation has been similarly reported in other models and is a key mechanism that explains muscle wasting. In this study, we observed a reduction in PI3K/Akt expression and an increase in the expression of MuRF, Atrogin-1, and myostatin expression in the muscles of aging rats. In contrast, the KGC01CE administration treatment increased the PI3K/Akt expression and decreased the expression of MuRF, atrogin-1, and myostatin in a dose-dependent manner. These results confirm that KGC01CE can improve age-related muscle wasting through enhancing protein synthesis and inhibiting degradation.

Muscle synthesis and degradation are one of the many molecular biological mechanisms, including antioxidation, muscle cell differentiation, energy metabolism, apoptosis, and anti-inflammation. Recent research has suggested that Ce water extract promotes muscle cell differentiation through increased Myf5 and myogenin and inhibits muscle wasting induced by AICAR via reducing MuRF [43]. Moreover, fermented Ce extract has been reported to regulate genes associated with muscle protein degradation (atrogin-1 and MuRF) and muscle fiber synthesis (MyoD and Myf5) to control muscle mass and strength [44,45,46,47]. In this reference, the fermentation of deer antler extract increased the content of sialic acid. The direct treatment of cells with sialic acid resulted in a significant reduction in atrogin-1 and MuRF. These results are consistent with the observed reduction in degrading proteins due to the KGC01CE treatment. Furthermore, changes in the expression of sialic acid, a major component of Ce extract, have been shown to induce muscle weakness and atrophy, with similar phenomena observed in age-related muscle wasting. Research on GNE myopathy and sialic acid has indicated that reduced sialylation and increased ROS generation occur, while external sialic acid intake increases overall sialylation, reducing ROS and protein S-nitrosylation [48]. Moreover, a decrease in the sialic acid levels in the body can lead to increased oxidative stress, which may induce muscle atrophy. Clinical studies that have compared young men (18–25 years, *n* = 8) and elderly men (72–78 years, *n* = 10) have shown a significant reduction in monomeric sialic acid in the elderly group, suggesting an association between sialic acid and skeletal muscle structure and function [49]. Eu water extract also significantly increased the muscle mass in aged mice via inhibiting the expression of caspase-3 [50]. Pinoresinol diglucoside, a major component of Eu extract, is known for its antioxidative and anti-inflammatory effects. Oxidative stress and inflammation are major causes of muscle wasting and can occur due to aging [51]. Based on these results, KGC01CE may be involved not only in muscle protein synthesis and degradation but also in addressing key causes of muscle wasting, such as muscle differentiation, oxidative stress, inflammation, and apoptosis.

To fully understand the impact of KGC01CE treatment on muscle wasting, further research is needed to determine how its physiologically active components affect muscle metabolism, oxidative stress, inflammation, protein synthesis/degradation, and cell apoptosis. Moreover, studies investigating the synergistic effects of these components and their interactions with other signaling pathways related to muscle health will be able to provide a deeper understanding of their therapeutic potential. KGC01CE is considered a safe dietary supplement with a long history of traditional use. Due to its diverse components, it may be effective against sarcopenia, a multifactorial disease, considering its multi-target effects on oxidative stress, inflammation, apoptosis, muscle differentiation, and energy metabolism. While the present study has shown promising results using animal models, it is essential to validate these findings in future human clinical trials. Thus, future research is needed to evaluate the impact of KGC01CE on muscle mass, strength, and overall physical function across various populations.

## 5. Conclusions

In this study, the KGC01CEtreatment was found to improve muscle atrophy in both cellular and animal experiments. The consumption of KGC01CE was found to significantly increase muscle mass, lean mass, grip strength, and muscle fiber CSA via activating the muscle synthesis pathway (PI3K/Akt pathway) and suppressing the degradation pathways (MuRF, atrogin, and myostatin-1) in aged rats. These findings suggest that KGC01CE could serve as a novel therapeutic strategy for muscle wasting or sarcopenia.

## Figures and Tables

**Table 1:** Animal experiment design.

Group	Experiment	*n*	Animal
NC	Saline	6	Young SD Rat (Male, 3 months)
C	Saline	6	Aged SD Rat (Male, 15 months)
KGC01CE – 50	CE(1:3) 50 mg/kg b.w	6	
KGC01CE – 100	CE(1:3) 100 mg/kg b.w	6	
KGC01CE – 200	CE(1:3) 200 mg/kg b.w	6	

NC: young SD Rats, C: aged SD Rats, KGC01CE − 50: aged SD Rats + CE(1:3) 50 mg/kg b.w, KGC01CE − 100: aged SD Rats +CE(1:3) 100 mg/kg b.w, KGC01CE − 200: aged SD Rats + CE(1:3) 200 mg/kg b.w.

**Table 2:** Serum biochemical analysis in different groups of aged rats after 12 weeks of treatment.

Group	AST	ALT	CREA	BUN
C	58.3 ± 4.8	16.3 ± 4.4	0.3 ± 0.1	7.7 ± 1.0
KGC01CE − 50	61.8 ± 11.8	17.0 ± 4.9	0.3 ± 0.1	7.6 ± 1.0
KGC01CE − 100	55.7 ± 20.2	17.0 ± 6.7	0.3 ± 0.2	6.8 ± 1.0
KGC01CE − 200	52.0 ± 9.5	13.3 ± 4.3	0.3 ± 0.1	7.3 ± 1.5

Data are expressed as mean ± standard deviation (*n* = 6). C: aged SD rats, KGC01CE − 50: aged SD rats + CE(1:3) 50 mg/kg b.w, KGC01CE − 100: aged SD rats +CE(1:3) 100 mg/kg b.w, KGC01CE − 200: aged SD rats +CE(1:3) 200 mg/kg b.w. ALT: alanine aminotransferase, AST: aspartate aminotransferase, BUN: blood urea nitrogen, CREA: creatinine.

## Data Availability

The data used in this study are available from the corresponding author upon request.

## Supplementary Materials

The following supporting information can be downloaded at: https://www.mdpi.com/article/10.3390/cimb46100664/s1. Figure S1. Effect of combinations of Ce and Eu ratios on muscle mass and grip strength in aged rats. (A) animal experimental design (*n* = 5), (B) GAS musle weight, and (C) grip strength. 15-month-old rats were orally administered ce, eu, CE(3:1), CE(1:1) and CE(1:3) at a concentration of 100 mg/kg for 12 weeks. A positive control was administered orally at 10 mg/kg for 12 weeks. NC: Young SD rats, C: aged SD rats, PC: aged SD rats + Oxymetholone, Ce: aged SD rats + Ce extract, Eu: aged SD rats + Eu extracts, CE(3:1): aged SD rats + Ce extract: Eu extract(3:1), CE(1:1): aged SD rats + Ce extract: Eu extracs(1:1), CE(1:3): aged SD rats + Ce extract: Eu extract(1:3), Data were expressed as mean ± standard deviation (*n* = 5). * *p* < 0.05, ** *p* < 0.01, n.s: not significant.
