# Supplementary material for: Protective Effects of Cervus elaphus and Eucommia ulmoides Mixture (KGC01CE) on Muscle Loss and Function in Aged Rats
Source: Curr Issues Mol Biol. 2024 Oct 4;46(10):11190–206. doi: 10.3390/cimb46100664 (PMC11506417; doi:10.3390/cimb46100664)
Supplement: Supplementary file 1 [file cimb-46-00664-s001.zip › cimb-3207342-supplementary.pdf]

current issues in molecular biology

MDPI

A

Group	Experiment	Animals
NC	Saline	Young SD Rat (Male, 3 months)
C	Saline	
PC	Oxymetholon 10 mg/kg b.w	
Ce	Ce 100 mg/kg b.w	
Eu	Eu 100 mg/kg b.w	Aged SD Rat (male, 15months)
CE(3:1)	Ce:Eu (3:1) 100mg/kg b.w	
CE(1:1)	Ce:Eu (1:1) 100 mg/kg b.w	
CE(1:3)	Ce:Eu (1:3) 100 mg/kg b.w	

B

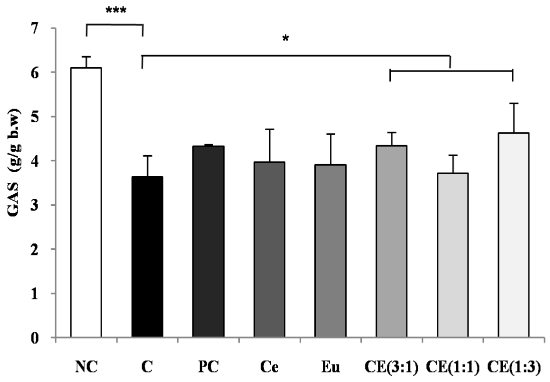

C

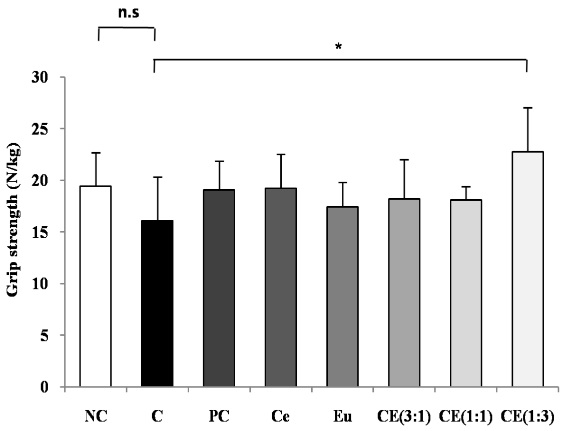

Figure S1. Effect of combinations of Ce and Eu ratios on muscle mass and grip strength in aged rats. (A) animal experimental design (n=5), (B) GAS musle weight, and (C) grip strength. 15-month-old rats were orally administered ce, eu, CE(3:1), CE(1:1) and CE(1:3) at a concentration of 100 mg/kg for 12 weeks. A positive control was administered orally at 10 mg/kg for 12 weeks. NC: Young SD rats, C: aged SD rats, PC: aged SD rats + Oxymetholone, Ce: aged SD rats + Ce extract, Eu: aged SD rats + Eu extracts, CE(3:1): aged SD rats + Ce extract : Eu extract(3:1), CE(1:1): aged SD rats + Ce extract : Eu extracs(1:1), CE(1:3): aged SD rats + Ce extract : Eu extract(1:3), Data were expressed as mean±standard deviation(n=5). *p< 0.05,** p <0.01, n.s: not significant.
